# Sensitive LC-MS/MS Assay for Total Testosterone Quantification on Unit Resolution and High-Resolution Instruments

**DOI:** 10.3390/jcm13237056

**Published:** 2024-11-22

**Authors:** Jill K. Wolken, Meghan M. Peterson, Wenjing Cao, Keith Challoner, Zhicheng Jin

**Affiliations:** 1 University of Wisconsin Hospital and Clinics, Madison, WI 53792, USA; 2 Department of Pathology and Laboratory Medicine, University of Wisconsin-Madison, 600 Highland Avenue, Madison, WI 53792, USA

**Keywords:** testosterone, multiple reaction monitoring, high-resolution mass spectrometry, assay harmonization, parallel reaction monitoring

## Abstract

**Background**: Testosterone is an androgenic hormone that plays important roles in both males and females. The circulating levels of total testosterone vary from 1 to 1480 ng/dL. High-throughput immunoassays often lack accuracy in lower concentration ranges (below 100 ng/dL), particularly when used for females or children. To address this limitation, we developed a total testosterone LC-MS/MS assay on three instruments. **Methods**: Sample preparation began with the dilution and conditioning of 200 µL of serum. A supported liquid extraction cartridge was used to extract the analyte from biological matrices. Chromatographic separation was achieved using a C18 column with a runtime of 5 min per sample. This assay was validated on a Triple Quad 6500 and an API 4500 instrument. **Results**: Method validation was carried out according to the CLSI C62-ED2 guideline and our hospital protocol. The within-day coefficient of variation (CV) was less than 10% and the between-day CV was less than 15%. The assay had a limit of quantitation of 0.5 ng/dL with an analyte measure range of 2–1200 ng/dL. A comparison using Deming regression and Bland–Altman plots showed that this assay correlated well with a reference method. The results from the API 4500 and an Orbitrap were consistent with those from the TQ 6500. Both serum-separator tubes (BD) and serum-activator tubes were found to be suitable. **Conclusions**: We successfully developed and validated a robust total testosterone LC-MS/MS assay for routine clinical testing. This assay was harmonized across two triple quadrupole instruments and one high-resolution mass spectrometer.

## 1. Introduction

Testosterone is a steroid hormone that is crucial in the development of male reproductive tissues and essential for the maintenance of secondary sexual characteristics. It can be synthesized from cholesterol or converted from precursors such as dehydroepiandrosterone (DHEA). In men, total testosterone measurement is important in hypogonadism workup. In women, testosterone serves as a precursor of estradiol and elevated circulating levels are associated with hirsutism, virilization, and oligomenorrhea. Most circulating testosterone is bound to sex hormone-binding globulin (SHBG) or albumin. Only a small fraction (about 2% to 3%) remains unbound and is the active form [1]. SHBG binds testosterone with very high affinity and albumin binds it with lower affinity. The unbound and albumin-bound fractions together make up approximately 35% of testosterone and represent the bioavailable form [2]. In children, total testosterone is used to evaluate precocious or delayed puberty.

Accurately measuring testosterone concentrations can be challenging due to the presence of structurally related steroid hormones, a wide concentration range, and low levels in females and children [3]. Previously, total testosterone was measured by a chemiluminescent microparticle immunoassay (CMIA) at our institution. It is a competitive immunoassay using anti-testosterone monoclonal-antibody-coated microparticles and acridinium-labeled testosterone. The analyte concentration is inversely related to the detected optical signal. The measurable range listed in the package insert is 13–1009 ng/dL (0.45–35 nmol/L). This assay is the appropriate test for adult males because their total testosterone levels are ≥230 ng/dL [1]. However, immunoassays suffer from low specificity and have difficulty in accurately measuring testosterone levels at concentrations less than 100 ng/dL [4]. Providers have requested us to develop a mass spectrometry-based total testosterone assay for female (reference range: 12–58 ng/dL) and pediatric patients [1,3].

Several studies have reported mass spectrometry methods for the quantitation of total testosterone in serum or plasma. In 1997, Tiller et al. introduced a LC-MS/MS method to quantify testosterone spiked plasma samples [5]. In 2003, Starcevic et al. published the first LC-MS/MS method for the measurement of endogenous testosterone levels in human serum [6]. Briefly, this method involved liquid–liquid extraction to enrich the analyte from two milliliters of serum. The reported analyte measurement range (AMR) was 50–2000 ng/dL on an API 300 triple quadrupole instrument. Two years later, Cawood et al. published a sensitive method that only used 50 µL of serum, extending the AMR to 7–2880 ng/dL and reducing the runtime to 5 min [7]. In 2008, Shiraishi et al. reported a sensitive method that quantified testosterone and dihydrotestosterone simultaneously, reducing the limit of quantitation (LOQ) to 2 ng/dL [8]. However, this method used a microflow HPLC system with the flow rate at 45 µL/min, and an analysis time of 18.5 min per sample, long for a routine clinical assay. In 2013, French et al. developed a sensitive method on a regular-flow HPLC system [9]. The assay linear range was 2–1263 ng/dL, with a sample volume of 200 µL. Liquid–liquid extraction was a conventional sample preparation technique used in these publications. 

Meanwhile, a clinical laboratory in Finland reported an automated online column-switching solid-phase extraction (SPE) method for testosterone quantification [10]. The analysis time was 2.7 min for the online SPE column and 6.7 min for chromatographic separation, with an AMR of 7–1008 ng/dL (0.25–35 nmol/L). A reference laboratory in the United States reported a high-turbulence flow HPLC, with the online SPE method achieving an assay linear range of 2 to 2000 ng/dL and a LOQ of 0.3 ng/dL [11]. The total analysis time was 4.5 min, which was further reduced to 1.2 min per sample using a multiplex LC system. In 2013, the Centers for Disease Control and Prevention published a reference measurement procedure, aiming to standardize testosterone measurements [12]. This method used a long linear gradient (16 min) to separate eighteen steroid hormones for interference testing. The AMR was 2.5–1000 ng/dL (0.09–34.7 nmol/L), requiring 0.5 mL of male serum and 1.0 mL of female serum.

Given its high sensitivity and specificity, LC-MS/MS has emerged as the preferred method for the quantification of steroid hormones in human serum [13,14]. Our goal is to develop a sensitive routine procedure for total testosterone measurement using LC-MS/MS instruments.

## 2. Materials and Methods

### 2.1. Reagents and Materials

Testosterone (1.0 mg/mL), epitestosterone (1.0 mg/mL), and [2,3,4-^13^C_3_]testosterone (100 µg/mL) were purchased from Cerillant (Round Rock, TX, USA). Ammonium hydroxide (99.99%) and dichloromethane (99.8%) were obtained from Sigma-Aldrich (St. Louis, MO, USA). Ultra-low steroid hormone serum was sourced from Golden West Biologicals (Temecula, CA, USA). Acetonitrile (99.9%), formic acid (99.0%), and water were from Fisher Scientific (Hampton, NH, USA). Support liquid extraction cartridge, Isolute SLE+ 400 µL, was procured from Biotage (Charlotte, NC, USA). 

### 2.2. Sample Preparation

Serum samples were brought to ambient conditions while preparing sample-conditioning solution (0.5 M ammonium hydroxide) and the internal standard (IS) working solution (1000 ng/dL, [2,3,4-^13^C_3_]testosterone in water). Two hundred microliters of serum samples were spiked with 25 µL of the IS solution and gently mixed with the sample-conditioning solution (200 µL). The mixture was transferred to an SLE+ 400 µL cartridge using a pipette. A positive pressure manifold (Biotage) was used to load the mixture onto the cartridge. Analytes were extracted twice using 900 µL of dichloromethane. The combined eluate was dried under nitrogen gas and resuspended in 120 µL of sample-reconstitution solution composed of 30% acetonitrile (*v*/*v*) in water. Processed samples were stored in a refrigerator prior to analysis. 

### 2.3. Standard and Quality Control

Calibrators and quality controls were prepared in-house by spiking testosterone standard solution into hormone-free serum. The calibrator concentrations were 2, 10, 50, 250, 500, and 1200 ng/dL. A standard from a different lot was purchased to prepare quality control samples at the concentrations of 35, 100, and 600 ng/dL. No difference was observed between lots. The quality controls were prepared along with unknowns on each batch to ensure that sample preparation and instrument performance worked as expected.

### 2.4. Unit Resolution LC-MS/MS Conditions

Liquid chromatography and mass spectroscopy conditions were optimized on an API 4500 (SCIEX, Framingham, MA, USA) and a Triple Quad 6500 (SCIEX) triple quadrupole instruments. Each system was interfaced with a 1260 HPLC (Agilent, Santa Clara, CA, USA). The analytical column was Restek Ultra C18, 5 µm, 50 × 2.1 mm (Restek, Bellefonte, PA, USA) maintained at 35 °C. Mobile phases consisted of 5% acetonitrile (*v*/*v*) and 0.1% formic acid in water (mobile phase A) and 95% acetonitrile (*v*/*v*) and 0.1% formic acid (mobile phase B). The gradient started at 40% mobile phase B, ramped to 80% B at 2.0 min, and to 95% B at 2.1 min. After holding at 95% B for 0.5 min, the column was equilibrated with 40% B for 2.4 min at a flow rate of 0.6 mL/min. Injection volume was 40 µL, with a runtime of 5 min per injection.

Both mass spectrometers were equipped with an electrospray ionization (ESI) source. Source parameters were consistent across instruments: source temperature at 550 °C, spray voltage at +5500 V, curtain gas flow at 40, and collision gas flow at 10. MRM transitions and parameters are shown in Table 1. Dwell time was 0.1 s with the total cycle time of 0.42 s. Each batch included a set of calibrator and QC samples. Data were acquired and processed using Analyst version 1.7 and MultiQuant version 3.0 (SCIEX) software. 

### 2.5. High-Resolution Mass Spectrometry (HRMS)

The Orbitrap Exploris 120 (Thermo Fisher Scientific, Waltham, MA, USA) was equipped with a Vanquish UHPLC. The LC parameters remain the same as described above. The ESI source temperature was maintained at 550 °C, with a spray voltage of +3400 V, sheath gas at 55, auxiliary gas at 5, sweep gas at 2, and the ion transfer tube at 350 °C. The acquisition method included one precursor scan followed by two product ion scans. The resolution was set at 120,000 for the precursor scan and 15,000 for the fragment ion scan, with normalized collision energy at 40%. Data acquisition and processing were performed using TraceFinder version 5.1 (Thermo Fisher Scientific).

### 2.6. Method Validation

This test was validated on API 4500 and TQ 6500 instruments. Four contrived specimens were used to evaluate within-day and between-day imprecisions. For within-day imprecision, four samples were extracted and analyzed 20 times within a day. For between-day studies, four samples were extracted in triplicate each day for ten days. Analytical precision was deemed acceptable if the CV was less than 15%. Calibrator accuracy should be within ±10% from theoretical values. QC results are expected to fall within three standard deviations of the established means. A split-sample comparison study was conducted to assess method accuracy by sending 39 samples to a reference laboratory using a mass spectrometry-based method. The LOQ was the minimum concentration at which the CV was within 20%. Carryover, matrix effect, and interference studies were performed according to established procedure and acceptance criteria [15]. Testosterone stability was assessed for serum specimens stored in a refrigerator (4 to 8 °C) or a freezer (−18 to −25 °C) for up to one month. Twenty-two specimens were collected to verify the reference range. The results from blood drawn in serum-separator tubes (BD, Franklin Lakes, NJ, USA) and clot-activator tubes (red-top) were compared to determine the acceptability of serum separator tubes. 

## 3. Results

### 3.1. Assay Development

**Figure 1:**
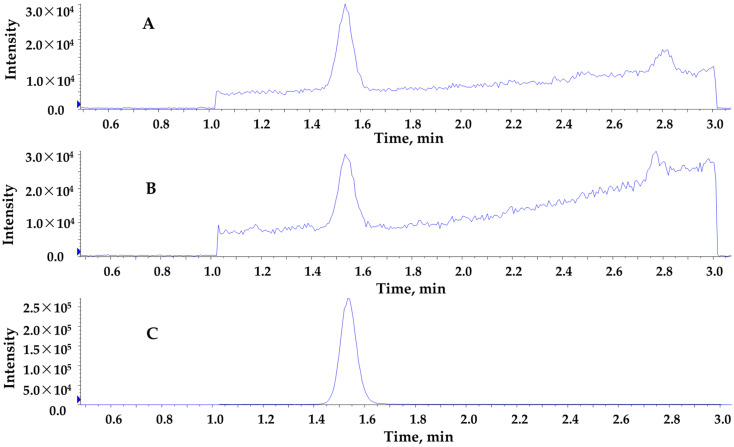
Extracted ion chromatograms of analyte in the lowest calibrator (2 ng/dL). Calibrators were prepared in hormone-free serum and extracted along with samples in each batch. (**A**) Quantifier ion pair, 289/97. (**B**) Qualifier ion pair, 289/109. (**C**) Internal standard transition, 292/100.

Testosterone is a small molecule with an average molecular weight of 288.4 Da. The product ion spectrum collected from a triple quadrupole instrument with an ESI source showed that the dominant fragment ions were *m*/*z* 97 and *m*/*z* 109 [14,15]. Both fragments were derived from the A ring and their possible structures were proposed previously [16,17]. We noticed that the signal-to-noise ratio of *m*/*z* 97 ion was slightly better and chose it as the quantifier ion. MRM conditions were found to be the same for API 4500 and TQ 6500 instruments (Table 1). The divert valve was set to collect data in the window of 1–3 min. Testosterone was eluted at 1.5 min, and examples of extracted ion chromatograms are shown in Figure 1 for the lowest calibrator, 2 ng/dL (0.069 nmol/L).

### 3.2. Validation Data 

#### 3.2.1. Sensitivity, Linearity, and AMR

Our goal was to develop a routine quantitation method with the lower AMR around 2 ng/dL [9]. We prepared contrived samples by spiking the analyte into blank serum at the target concentrations of 0.5, 1, 2, 3.5, and 10 ng/dL. Specimens were extracted three times and analyzed on two unit-resolution instruments. The percentage CV was within 20% on API 4500 and TQ 6500, demonstrating that LOQ was ≤0.5 ng/dL. After reviewing the literature, we decided to set the upper AMR at 1200 ng/dL. A calibration curve is shown in Supplementary Figure S1, demonstrating a linear dynamic range of 2 to 1200 ng/dL.

#### 3.2.2. Imprecision

Within-day and between-day studies were conducted to assess the assay variation and stability over time. As shown in Table 2, the within-day CV was less than 6% and the between-day CV was ≤12%. These data demonstrated that the analytical precision met the acceptance criteria (CV ≤ 15%) on TQ 6500 and API 4500 instruments.

#### 3.2.3. Method Comparison

**Figure 2:**
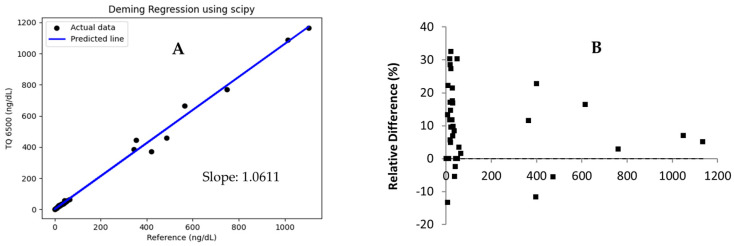
A comparison between the new assay on TQ 6500 and a reference method. (**A**) Deming regression plot generated using SciPy. (**B**) Bland–Altman plot.

Thirty-nine specimens across the AMR were tested in-house, and split samples were sent to a reference laboratory to assess the method accuracy. Deming regression and a Bland–Altman plot showed that the new assay on TQ 6500 correlated well with the reference method (Figure 2). The comparison results exhibited less than 7% positive bias, which falls within the acceptable criteria of ±10% bias. Because the test was intended for patients with testosterone levels likely to be under 100 ng/dL, a method comparison was carried out at this range (N = 31). As depicted in Supplementary Figure S2, the results indicated a positive bias of <4%, meeting the acceptable criteria.

#### 3.2.4. Carryover, Interference, Matrix Effect, and Storage Stability

Assay carryover was evaluated by injecting low QC after high QC samples according to the CLSI C62-ED2:2022 guideline. The carryover error was less than 0.05 ng/dL and below 25% of the lower limit of the measuring interval (LLMI). Endogenous interference studies analyzing five serum specimens showed that hemolysis (at 1000 mg/dL of hemoglobin) or icterus (at 40 mg/dL of bilirubin) did not affect the quantitation results (Supplementary Table S1). However, a lipemic sample in the presence of triglyceride-rich lipoprotein at 1500 mg/dL caused a negative bias that was greater than 20%. As a result, the gross lipemic sample would be rejected. The epitestosterone peak was present at 2.1 min, which was separated from the testosterone peak and did not interfere with quantitation (Supplementary Figure S3). A post-column infusion experiment confirmed that ion suppression did not affect quantitation (Supplementary Figure S4). Human serum samples were found to be stable for up to 14 days when stored in a refrigerator and 30 days in a freezer (Supplementary Figure S5). The characteristics of this method are summarized in Table 3.

### 3.3. High-Resolution Mass Spectrometry

**Figure 3:**
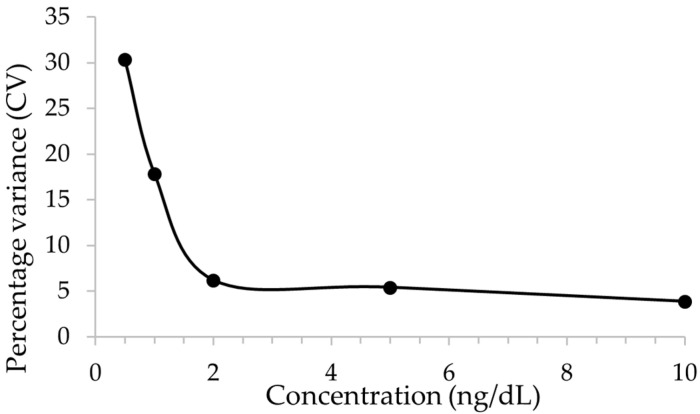
Determination of limit of quantitation (LOQ) on OE120.

We transferred this method to a high-resolution mass spectrometer, Orbitrap Exploris 120 (OE120). The LC parameters are kept the same as those on two triple quadrupole instruments. Testosterone peak was present at 1.1 min on the Vanquish UHPLC. The OE120 is a hybrid ion trap instrument, and full scan spectra were collected for precursor ion and product ion scans. The quantitation method was built in parallel reaction monitoring (PRM) mode. An example of product ion spectrum is shown in Supplementary Figure S6. The accurate mass of the precursor and product ions are shown in Supplementary Table S2. The LOQ on the OE120 was found to be 1 ng/dL (Figure 3). The testosterone assay remained linear in the range of 2–1200 ng/dL.

### 3.4. Multiple Instrument Comparison

**Figure 4:**
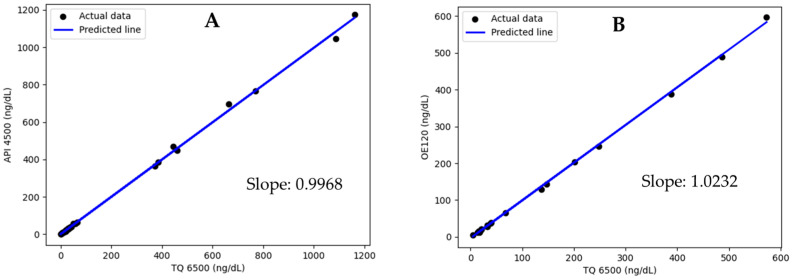
Method comparison using scatter plot and Deming regression analysis. (**A**) API 4500 vs. TQ 6500. (**B**) OE120 vs. TQ 6500.

TQ 6500 was the primary instrument for this assay. It is necessary to validate this assay on another instrument, API 4500, to prepare for unexpected instrument downtime. Scatter plot and Deming regression analysis (N = 39) showed that results from both instruments correlated well with the slope of 0.9968 (Figure 4A). The accuracy of this method on high-resolution mass spectrometry, OE120, was evaluated against TQ 6500. Deming regression using SciPy (N = 20) showed a slope of 1.0232 with a correlation R of 0.9996 (Figure 4B). In comparing the three instruments, the slopes fell within the acceptance range of 0.9 to 1.1. This result shows no significant difference in the testosterone assay developed on either the unit resolution or high-resolution Orbitrap instrument.

### 3.5. Reference Range Verification 

We adopted testosterone reference intervals that were used by the reference laboratory, which were published previously [18]. Prior to the test going live, we recruited apparently health female volunteers (N = 22) who graciously donated blood for reference range verification. Blood was collected in red-top and serum-separator tubes (SSTs) to carry out a tube-type comparison study. The total testosterone levels of 91% female volunteers were within the reference range of 9–55 ng/dL. As Student’s *t*-test analysis revealed no significant difference between serum samples collected in SSTs and red-top tubes (*p* = 0.838), both tube types were deemed acceptable.

## 4. Discussion and Conclusions

Mass spectrometry is the gold-standard method for steroid hormone measurements [19]. Testosterone is an excellent example for demonstrating the advantages of a mass spectrometry-based method in sub-concentration ranges (<100 ng/dL). However, developing a testosterone assay remains challenging due to the presence of steroid hormone inferences, a wide concentration range, and protein binding. Sample preparation often involves multiple steps, with liquid–liquid extraction being a common approach. Recently, SPE and supported liquid extraction (SLE) have become more popular, because these tools facilitate process automation and increase throughput. We achieved reproducible results using a SLE cartridge and validated the assay using a positive pressure manifold to reduce manual steps [20]. A key step in clinical assay development is to determine the lower and upper limit of the measurement interval (ULMI). After reviewing the literature, we decided to set the LLMI at 2 ng/dL and the ULMI at 1200 ng/dL. We validated this method on both TQ 6500 and API 4500 instruments. 

High-resolution mass spectrometry is relatively new to clinical laboratories, and its performance has not been well characterized [21]. OE120 offers three options for quantitative assay development. An acquisition method can be developed, utilizing (1) precursor ion intensity in full scan, (2) precursor ion intensity in selected ion monitoring (SIM), (3) parallel reaction monitoring (PRM), i.e., fragment ion intensity in product ion scan. In theory, the PRM is comparable to the MRM on triple quadrupole instruments. We developed a quantitation method using the PRM approach and assessed the instrument performance. The assay sensitivity, linear dynamic range, and accuracy met the acceptance criteria. Ongoing work aims to validate this assay on the OE120 instrument. These results demonstrated that this method was harmonized across all three instruments (Figure 4). Clinical assay harmonization and standardization on multiple LC-MS/MS instruments can be challenging. In 2008, the Centers for Disease Control initiated hormone standardization project (HoSt) and published reference procedures for testosterone and estradiol assays [4,19]. The reference method depicted in Figure 2 originated from a HoSt-certified reference laboratory. To continuously monitor assay accuracy, we obtained the accuracy-based survey samples from the CAP. Since going live in 2022, this assay has consistently met dual evaluation criteria, i.e., within ±20% of peer group mean and accuracy-based targets.

Our method offers several advantages: (1) chromatography conditions were tested on both HPLC and UHPLC systems; (2) sample preparation was partially automated using a positive pressure manifold, which significantly increased throughput; (3) the analytical method is fast, sensitive, and robust, fulfilling the requirements of a routine clinical assay. However, the limitation is that DHEA was not tested as a potential endogenous interference. Additionally, the chromatographic separation time could be further reduced on the UHPLC system.

In summary, we developed a sensitive LC-MS/MS assay for total testosterone quantitation. This assay was validated on both API 4500 and TQ 6500 instruments for routine clinical testing. The sensitivity, linear dynamic range, and accuracy of this assay on one high-resolution mass spectrometer met the established acceptance criteria.

## Figures and Tables

**Table 1:** MRM transitions and parameters for API 4500 and TQ 6500.

	Precursor (*m*/*z*)	Fragment (*m*/*z*)	EP ^0^ (v)	DP ^1^ (v)	CE ^2^ (v)	CXP ^3^ (v)
Quantifier ion	289	97	10	96	33	12
Qualifier ion	289	109	10	96	33	8
IS 1	292	100	10	96	33	12
IS 2	292	112	10	96	33	8

^0^: entrance potential; ^1^: declustering potential; ^2^: collisional energy; ^3^: collision-cell exit potential.

**Table 2:** Total testosterone within-day and between-day variations on two instruments.

	Instrument	Mean (ng/dL)	CV (%)
Within-day (N = 20)	TQ 6500	9.2	1.6
		33.6	1.6
		107.5	1.5
		633.6	1.4
	API 4500	8.9	2.2
		35.7	1.7
		100.0	1.8
		522.5	5.5
Between-day (N = 30, 10 days)	TQ 6500	9.7	10.9
		35.6	5.6
		130.3	7.1
		649.5	4.3
	API 4500	10.0	11.0
		36.4	3.7
		132.2	5.4
		657.5	4.7

**Table 3:** Method performance summary on triple quadrupole instruments.

Characteristic		Specification
Specimen type		Red-top; serum-separator tube
Minimum volume		0.5 mL
AMR		2–1200 ng/dL (0.069–41.5 nmol/L)
LOQ		0.5 ng/dL (0.017 nmol/L)
Interference	Hemolysis	OK
	Icterus	OK
	Gross lipemia	Rejected
Storage stability	Refrigerator	14 days
	Frozen	30 days

## Data Availability

The original contributions presented in the study are included in the article/Supplementary Material, further inquiries can be directed to the corresponding author.

## Supplementary Materials

The following supporting information can be downloaded at: https://www.mdpi.com/article/10.3390/jcm13237056/s1, Figure S1: A six-point calibration curve with concentrations of 2, 10, 50, 250, 500, and 1200 ng/dL. Figure S2: Method comparison at a low concentration range (<100 ng/dL) using Deming regression. Figure S3: Extracted ion chromatogram demonstrating the baseline separation of testosterone and epitestosterone. Figure S4: Matrix effect study by post-column infusion on TQ 6500. Figure S5: Sample stability study after storage in refrigerator for 14 days (A) or in freezer for 31 days (B). Figure S6: High-resolution testosterone MS^2^ spectrum acquired on OE120. Table S1: Hemolysis, icterus, and lipemia interference studies. Table S2: Accurate mass of precursor and fragment ions on the OE120.
