# Supplementary material for: Sensitive LC-MS/MS Assay for Total Testosterone Quantification on Unit Resolution and High-Resolution Instruments
Source: J Clin Med. 2024 Nov 22;13(23):7056. doi: 10.3390/jcm13237056 (PMC11641924; doi:10.3390/jcm13237056)
Supplement: Supplementary file 1 [file jcm-13-07056-s001.zip › jcm-3291211-supplementary.pdf]

Supplementary Data

# Sensitive LC-MS/MS Assay for Total Testosterone Quantification on Unit Resolution and High-Resolution Instruments

**Jill K. Wolken [1], Meghan M. Peterson [1], Wenjing Cao [1,2], Keith Challoner [1], and Zhicheng Jin [1,2]**

[1] University of Wisconsin Hospital and Clinics, Madison, WI, USA
[2] Department of Pathology and Laboratory Medicine, University of Wisconsin – Madison, WI, USA

Corresponding author:
Zhicheng Jin
Department of Pathology and Laboratory Medicine
University of Wisconsin – Madison
600 Highland Avenue, Madison, WI, 53792, USA.
E-mail: zjin237@wisc.edu (Z. Jin);
Tel.: +1 (608) 263-7508

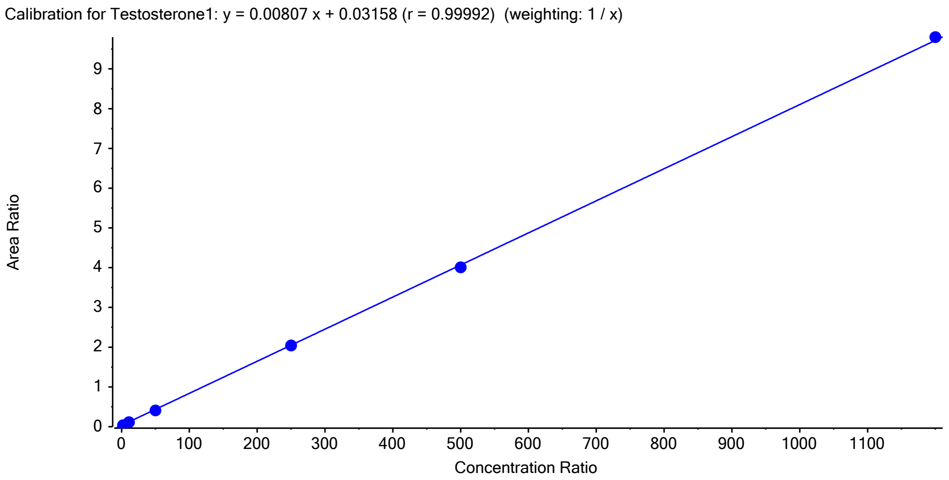


**Supplementary Figure S1**. A six-point calibration curve with concentrations of 2, 10, 50, 250, 500, and 1200 ng/dL.

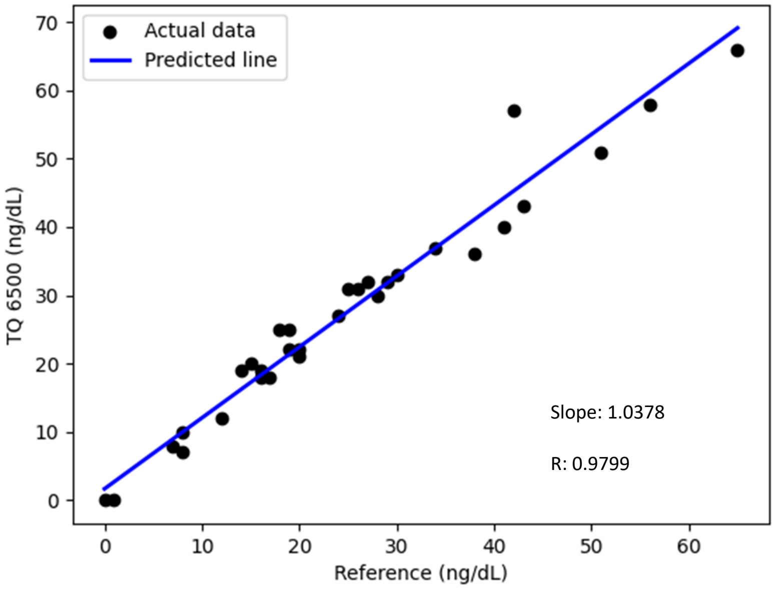


**Supplementary Figure S2**. Method comparison at a lower concentration range (< 100 ng/dL) using Deming Regression.

**Supplementary Table S1.** Hemolysis, icterus, and lipemia interference studies

| Category | Sample ID | Control (ng/dL) | Spiked (ng/dL) | Absolute Difference (ng/dL) | Relative Difference (%) |
|---|---|---|---|---|---|
| Hemolysis | S1 | 76 | 78 | 2 | 2.6 |
| | S2 | 658 | 641 | -17 | -2.6 |
| | S3 | 54 | 56 | 2 | 3.7 |
| | S4 | 56 | 57 | 1 | 1.8 |
| | S5 | 2 | 3 | 1 | 50 |
| | | | | | |
| Icterus | S6 | 24 | 23 | -1 | -4.2 |
| | S7 | 9 | 9 | 0 | 0 |
| | S8 | 27 | 26 | -1 | -3.7 |
| | S9 | 233 | 234 | 1 | 0.4 |
| | S10 | 99 | 99 | 0 | 0 |
| | | | | | |
| Lipemia | S11 | 52 | 45 | 7 | -13.5 |
| | S12 | 458 | 374 | 83 | -18.1 |
| | S13 | 16 | 15 | 1 | -6.3 |
| | S14 | 117 | 91 | 26 | -22.2 |
| | S15 | 82 | 77 | 5 | -6.1 |

**Supplementary Table S2**. Accurate mass of precursor and fragment ions on OE120

| Name | Precursor | Fragment | CE |
|---|---|---|---|
| Quantifier ion | 289.2162 | 97.0648 | 40 |
| Qualifier ion | 289.2162 | 109.065 | 40 |
| IS | 292.2263 | 100.0754 | 40 |

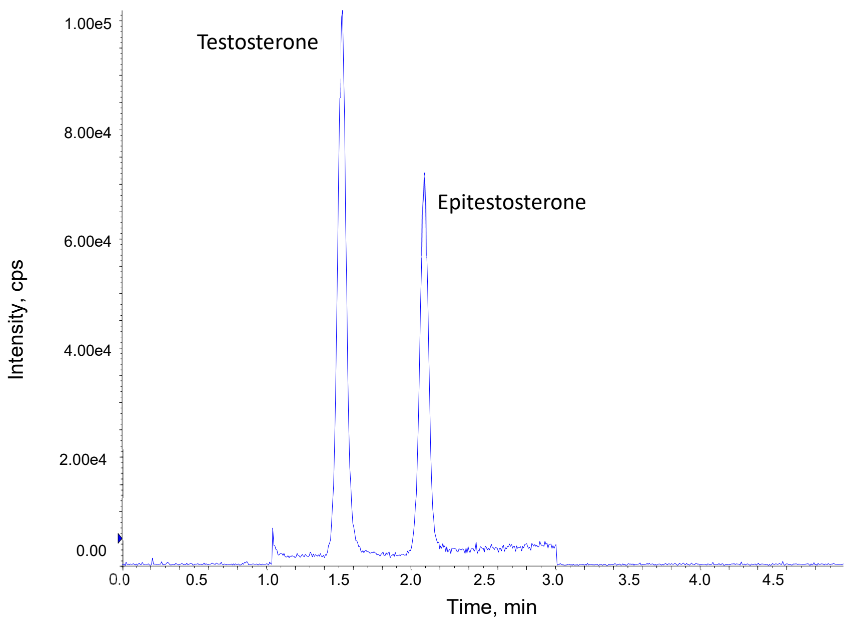


**Supplementary Figure S3**. Extracted ion chromatogram demonstrating the baseline separation of testosterone and epitestosterone.

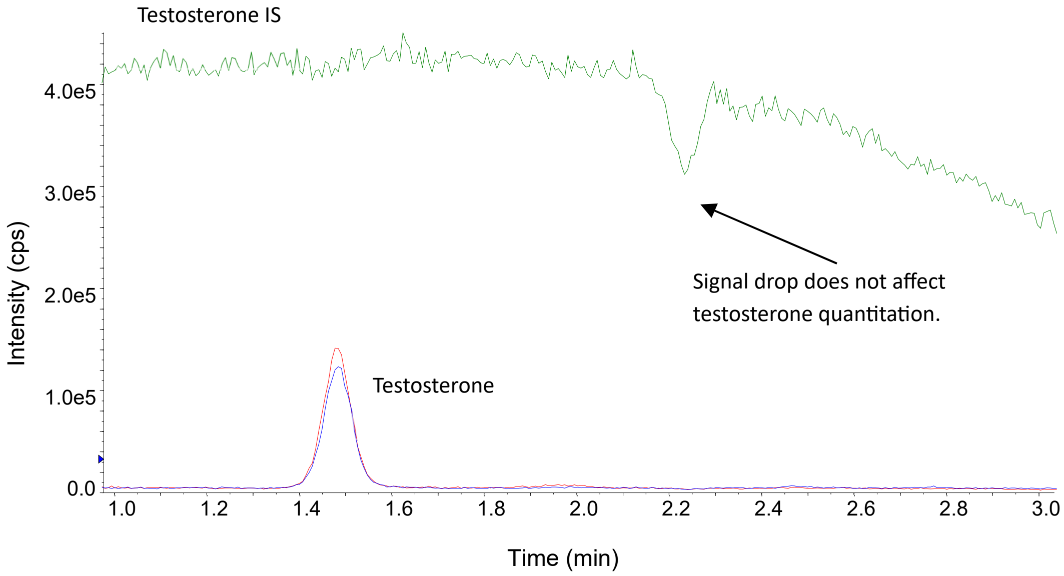


**Supplementary Figure S4**. Matrix effect study by post-column infusion on TQ 6500

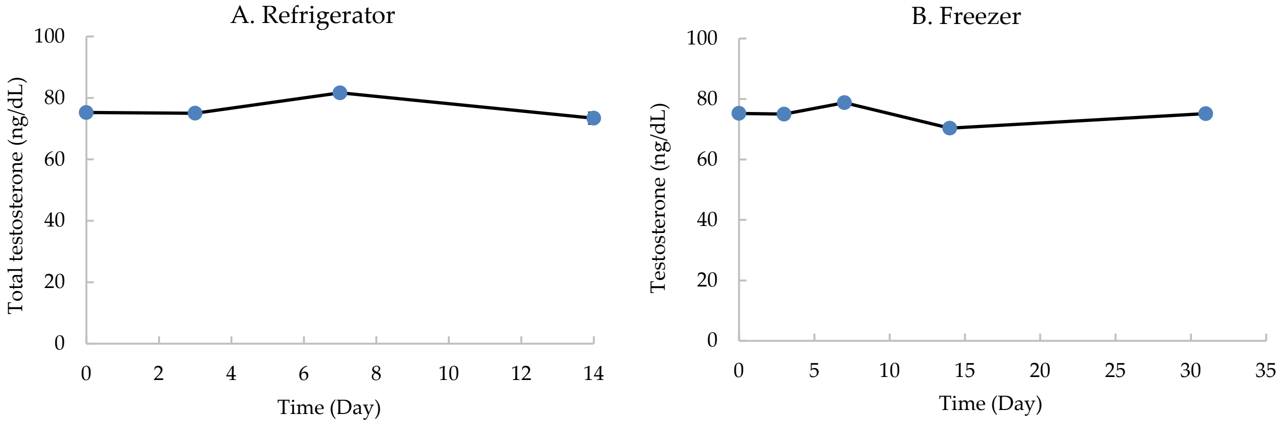


**Supplementary Figure S5**. Sample stability study after storage in refrigerator for 14 days (A) or in freezer for 31 days (B)

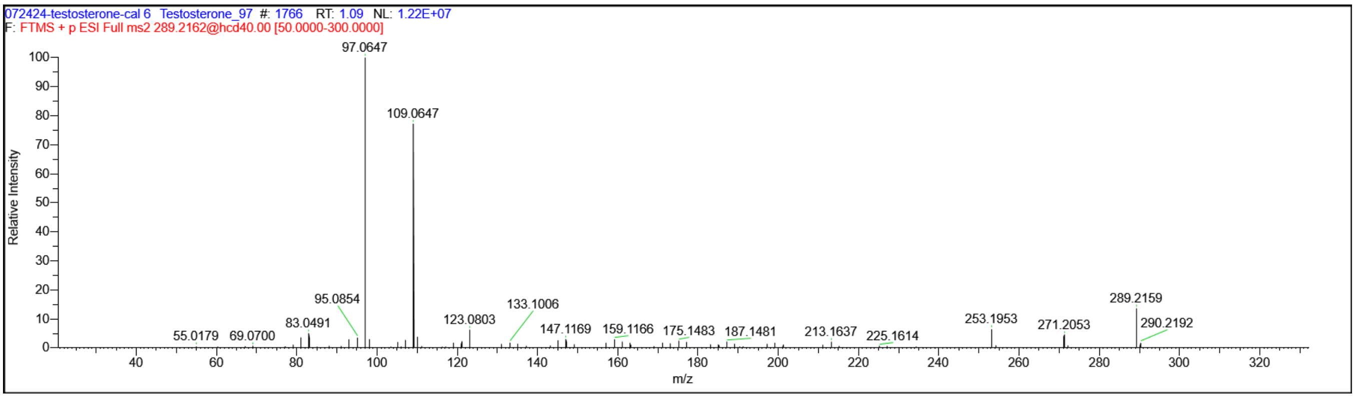


**Supplementary Figure S6.** High-resolution testosterone $MS^2$ spectrum collected on OE120.
